# Editorial: Factors Affecting Performance and Recovery in Team Sports: A Multidimensional Perspective

**DOI:** 10.3389/fphys.2022.877879

**Published:** 2022-03-23

**Authors:** Athos Trecroci, Damiano Formenti, Jason Moran, Dino Pedreschi, Alessio Rossi

**Affiliations:** ^1^Department of Biomedical Sciences for Health, Università degli Studi di Milano, Milan, Italy; ^2^Department of Biotechnology and Life Sciences, University of Insubria, Varese, Italy; ^3^School of Sport, Rehabilitation and Exercise Sciences, University of Essex, Colchester, United Kingdom; ^4^Department of Computer Science, University of Pisa, Pisa, Italy

**Keywords:** recovery, fatigue, soccer, wellbeing, cognition, perceptual response, rugby, physical performance

In a team sports season, players likely experience congested fixture schedules, characterized by multiple games within a short timeframe (Carling et al., 2015). To face such a dense modern competitive schedule, players often undergo a high number of training sessions. The combination of multiple games and numerous training sessions within a short time could induce marked psychophysiological stress on an athlete, making recovery between competitive events a crucial element in the training process (Doeven et al., 2018; Silva et al., 2018). Indeed, congested fixture schedules typical of a team sports season can greatly affect the recovery process between the events, thus preventing athletes from attaining optimal performance levels (Trecroci et al., 2020b). A condition of prolonged fatigue might be reflected on the overall psychophysiological status of the athlete, causing neuromuscular and biochemical perturbations as well as physical and cognitive performance declines and technical skill impairments alongside an increased likelihood of injury occurrence (Dupont et al., 2010). As performance and recovery in team sports depend on several factors (physical, technical, physiological, psychological, cognitive, and morphological), advancing knowledge on this issue should be based on a multidimensional approach.

In this context, the present Research Topic extends knowledge on the factors affecting sport performance and recovery, emphasizing the use of novel strategies to alleviate the potential carryover effects of fatigue. This Research Topic addresses this theme with the contribution of ten original research articles and two reviews. Nine of the Research Articles focused on soccer, and one on rink hockey. One review focused on quantifying fatigue in rugby and one narrative review assessed the effect of cannabidiol supplementation for the enhancement of recovery. Moreover, four articles evaluated the change in performance relating to different training approaches such as situations external to sport (COVID-19 lockdown) and players' neuromuscular status, while eight articles investigated athletes' recovery status in relation to training methodologies and intensity, match and player characteristics and drug supplementation effects.

The detraining effect caused by the COVID-19 outbreak were investigated by Souza et al.. These authors evaluated the effect of the suspension of training on physical performance in soccer players competing in Spain's *La Liga*. In particular, the researchers compared players' running patterns before and after the lockdown period (8 weeks), reporting that the total running distance and the high intensity running performance of professional soccer teams was maintained after the resumption of the competition. Interestingly, the number of substitutions and match duration significantly increased after the lockdown period in comparison to the previous season.

Two articles described the effect of different training programmes on sport performance. Sariati et al. assessed the effect of a 6-week change of direction (CoD) training intervention on dynamic balance, horizontal jump, speed, and CoD with and without the ball in youth male soccer players with different levels of maturity status (measured relative to peak height velocity). The authors found that the CoD training program improved balance, horizontal jump, and CoD without the ball in male preadolescent and adolescent soccer players. Interestingly, a greater improvement was detected in the post-peak height velocity players compared to the pre-peak height velocity players. Accordingly, Sariati et al. suggested that peak height velocity should be considered when programming CoD training for soccer players. Alternatively, Koral et al. investigated the effect of three different preseason training programs (i.e., plyometric, sprint interval, and small-sided games training) on recreationally trained soccer players' physical performance (i.e., sprint ability, CoD, and maximal aerobic speed). In this study, the authors demonstrated that the most effective training programme was sprint interval training, followed by plyometric training, while the players that performed the small-side games training obtained the lowest performance improvement.

Pleša et al. investigated the association between bilateral deficit in the countermovement jump and sprint, bilateral and single-leg jump, and CoD in volleyball players. Small to moderate correlations of lower limb bilateral deficit with CoD, sprint ability, and jump performance were revealed, suggesting that these players' performance characteristics should be useful in programming the amount of training time that each volleyball player should dedicate to unilateral lower-limbs training.

Within the present Research Topic, particular emphasis was also given to the study of recovery, as part of the complex training process. Trecroci et al. sought to establish the impact of two different post-match training interventions on the recovery timeframe of both perceptual (muscle soreness) and biochemical parameters (e.g., creatine kinase) after a soccer match. In this study, the authors employed an active recovery (AR) or soccer-specific training sessions (SST) protocol on the second day after match performance. The researchers observed a higher restoration of muscle soreness and creatine kinase in AR compared with SST within 72 h post-match timeframe. This result provides additional and novel data that may aid practitioners' decision-making process when two consecutive games are played within a 3-day period. From a practical perspective, a low dose of high-intensity training (i.e., AR) performed 48 h after a game may be less detrimental *per se* for subsequent exercise performance.

Silva et al. analyzed variations over a 6-week period of short-duration maximal jumping performance in professional soccer players exposed to different accumulated training loads and matches. The authors found that match participation was the main factor influencing countermovement jump (CMJ) performance. Specifically, it was observed that the sum of weekly training session loads of high metabolic load distance, accelerations/decelerations and total distance covered by the players were the best predictors of the CMJ performance variation. This result provides coaches and practitioners with additional and extended knowledge on the importance of monitoring and managing GPS-based metrics linked to high-intensity demand activities (i.e., accelerations) weekly. This could help them to implement strategies to increase players' readiness to play.

Fernàndez et al. provided an integrative approach to external (based on a local positioning system) and internal load (perceived exertion) dynamics for monitoring fitness and fatigue status in elite rink hockey players during a standard periodised microcycle. The authors assessed the differences between training sessions and matches and the potential association between the external and internal load metrics (including distance covered, accelerations/decelerations, and high-speed skating). It was found that the training sessions 3 and 2 days prior to the nearest match demonstrated the greatest external and internal loads within the microcycle (also compared with the corresponding match loads) by exhibiting an inverted “U-shaped” load dynamic across the training period. Moreover, the authors reported moderate-to-large associations between volume-related variables (i.e., distance covered) and perceived exertion and low correlation between high-intensity-related (i.e., high-speed skating) variables and perceived exertion. This data facilitates a deeper understanding of the load distribution (internal and external loads during trainings and the weekly match) within a regular elite rink hockey team microcycle, providing practical guidelines for managing the weekly training programme.

The review by Naughton et al. dealt with post-match fatigue and recovery in rugby players competing in different events (i.e., rugby league, rugby union, and rugby sevens). The authors' intent was to better explore the recovery dynamics of neuromuscular (e.g., CMJ), biochemical (e.g., creatine kinase), and self-reported (e.g., muscle soreness) measures along with the association between match-related fatigue metrics due to collisions and high-intensity locomotor actions. Their findings revealed the presence of acute (up to 24 h post-match), residual (from 24 to 72 h) and persistent (beyond 72 h) “windows of fatigue” in which players experience a progressive change in performance, biochemical, and subjective recovery. Moreover, the authors highlighted how such recovery time course strongly relates to the frequency and intensity of collisions during a match. Altogether, these findings shed a light on the importance of quantifying post-match recovery in rugby under a multidimensional perspective to embrace the aggregate match-related carryover effects of collisions and high-intensity locomotor actions. Rugby players (regardless of competitive events played) would likely benefit from sufficient time to recover and return to their pre-match level of conditioning.

The narrative review by Rojas-Valverde focused on the potential role of cannabidiol (CBD) as an ergogenic aid to promote better recovery between efforts of training sessions and competitions. The recent removal of CBD from the list of prohibited substances from the World Anti-Doping Agency has increased both its use in sport professionals and the study of its properties. Although the paucity of literature on this issue, CBD was demonstrated to have properties to boost exercise recovery as an anti-inflammatory, neuroprotective, analgesic, anxiolytic, and pain reliever. This evidence supports the potentiality of CBD to be used as a strategy to improve the efficacy and efficiency of recovery processes during exercise and to offset sport-related fatigue. However, considering also the lack of studies in elite athletes, the review emphasized the call for additional studies to explore the underlying physiological mechanisms related to the use of CBD in the field of sports science.

One experimental study proposed a novel questionnaire to assess the types of recovery practices utilized in team sport (Querido et al.). Although practices to mitigate fatigue and improve recovery are widely known, few studies have investigated the types of recovery methods used in team sports and the underlying reasons for these choices by medical and technical staff. The authors of this study developed a valid and reliable online questionnaire to examine the practices adopted in the 72 h post-match period in soccer teams. The questionnaire was proposed in the Portuguese language but can be used as a basis for increasing the knowledge of the current recovery practices in team sports after appropriate validation in a specific language.

Ishida et al. investigated seasonal changes in training load, neuromuscular performance, subjective recovery, and stress status and examined the relationship between training load and neuromuscular changes in National Collegiate Athletic Association (NCAA) female soccer players. Long-term strategic training plans are necessary to maximize neuromuscular performance throughout the competitive season. The main findings of this study showed that neuromuscular performance gradually increased from pre-season to the competitive period. This was accompanied by a decrement in training load metrics. Significant negative correlations were observed for weekly total distance with CMJ height and peak power, while positive correlations were observed for player load and CMJ height. These findings highlight the importance of quantifying the summer, pre-season, and in-season training loads together with neuromuscular performance in female competitive soccer players.

The study by Bian et al. focused on a current hot topic within the sports-science literature (i.e., mental fatigue and physical performance). The negative effect of a mentally fatiguing task on physical performance in soccer is well-established (Coutinho et al., 2018; Smith et al., 2018; Trecroci et al., 2020a). Most studies investigating the effect of mental fatigue on physical performance utilized a computerized cognitive task for inducing mental fatigue, though this method is considered to have poor ecological validity. In their study, the authors proposed a novel motor task requiring soccer-specific skills (i.e., 20-min repeated interval Loughborough Soccer Passing Test, LSPT) for inducing mental fatigue in soccer as compared to computerized cognitive task. The 20-min repeated interval LSPT, as a soccer-specific motor task, induced subjective mental fatigue similar to that of the 20-min Stroop task. The mental fatigue induced by the repeated interval LSPT induced a similar detrimental effect as the 20-min Stroop task on cognitive and soccer-specific skill performance. These findings supported the use of the 20-min repeated interval LSPT as an ecological task to induce mental fatigue in soccer.

In conclusion, the articles published within this Research Topic contribute to advance knowledge for a better understanding of sport performance and recovery factors in team sports. Their findings highlight the complexity behind the interaction of training, competition, performance, and recovery. We hope that this Research Topic will stimulate further research in this area. Future studies should further advance knowledge on the topic of sports performance and recovery by striking a balance between the strict scientific rigor of the experimental setting (considering internal and external validity) and the high level of applicability required for team sports (helping practitioners to manage training load metrics) using a multidimensional approach. For example, within an ecological approach, further studies will have to focus on in-season training protocols to understand how different training strategies between multiple weekly matches may affect fatigue, by a combination of physiological and performance adaptations in the long term. Finally, we thank all the authors, reviewers and editors for their valuable contributions to this Research Topic.

## Author Contributions

AT, AR, and DF wrote the original draft of this editorial, while DP and JM reviewed and edited it. All authors have approved the final version of this editorial.

## Funding

This work is supported by the European Community's H2020 Program under the Funding Scheme H2020-INFRAIA-2019-1 Research Infrastructures Grant Agreement 871042, www.sobigdata.eu, accessed on 2 November 2021, SoBigData++: European Integrated Infrastructure for Social Mining and Big Data Analytics. The funders had no role in study design, data collection and analysis, decision to publish, or preparation of the manuscript.

## Conflict of Interest

The authors declare that the research was conducted in the absence of any commercial or financial relationships that could be construed as a potential conflict of interest.

## Publisher's Note

All claims expressed in this article are solely those of the authors and do not necessarily represent those of their affiliated organizations, or those of the publisher, the editors and the reviewers. Any product that may be evaluated in this article, or claim that may be made by its manufacturer, is not guaranteed or endorsed by the publisher.
